# Mass cytometry reveals the corneal immune cell changes at single cell level in diabetic mice

**DOI:** 10.3389/fendo.2023.1253188

**Published:** 2023-09-05

**Authors:** Limin Qin, Qian Li, Liqiang Wang, Yifei Huang

**Affiliations:** ^1^ Department of Ophthalmology, The Third Medical Center, Chinese People's Liberation Army of China General Hospital, Beijing, China; ^2^ Department of Ophthalmology, The First Medical Center, Chinese People's Liberation Army of China General Hospital, Beijing, China; ^3^ Department of Ophthalmology, Medical School of Chinese People’s Liberation Army (PLA), Beijing, China

**Keywords:** tissue-resident memory T, myeloid-derived suppressor cells, γδ T cells, immune microenvironment, hyperglycemia

## Abstract

**Introduction:**

Diabetic ocular complications include sight-threatening consequences and decreased corneal sensitivity, characterized by decreased tear production, corneal sensitivity and delayed corneal epithelial wound healing. The pathogenesis of diabetic corneal disorders remains largely unknown. Growing evidence implies the participation of immune cells in the development of diabetic corneal diseases. Nonetheless, the immunological changes that result in diabetic corneal problems are largely unknown.

**Methods:**

Mass cytometry by time of flight (CyTOF) was used to investigate immune cell cluster alterations associated with diabetic corneal disorders. CyTOF test was performed on corneal cells at a single level from 21-week-old diabetic (db/db) and non-diabetic (db/m) mice. A panel of 41 immune-related markers monitored different immune cell types in diabetic corneas. To investigate the proportion of each immune cell subpopulation, an unsupervised clustering method was employed, and T-distributed stochastic neighbor embedding was used to visualize the distinctions between different immune cell subsets.

**Results:**

Through CyTOF test, we identified 10 immune cell subsets in the corneal tissues. In a novel way, we discovered significant immune alterations in diabetic corneas, including pronounced alterations in T cells and myeloid cell subgroups in diabetic corneas linked to potential biomarkers, including CD103, CCR2, SiglecF, Ly6G, and CD172a. Comprehensive immunological profiling indicated remarkable changes in the immune microenvironment in diabetic corneas, characterized by a notable decrease in CD103+CD8+ tissue-resident memory T (T_RM_) cells and Tregs, as well as a dramatic increase of γδT cells and subsets of CD11b+Ly6G+ myeloid-derived suppressor cells (MDSCs).

**Conclusion:**

CyTOF analysis revealed significant alterations in the immune microenvironment during the development of diabetic corneal complications. This study mapped the immune microenvironment landscape of type 2 diabetic corneas, providing a fundamental understanding of immune-driven diabetic corneal disorders.

## 1 Introduction

Even though up to two-thirds of diabetic patients suffer from ocular surface complications throughout their diabetic periods (1), the complications in the cornea, conjunctiva, and lacrimal gland have been poorly understood. The cornea, particularly its epithelium and nerve fibers, is an important site of injury due to persistent hyperglycemia (1). Patients with diabetes exhibit a progressive decrease in cornea nerve density and sensitivity, resulting in compromised corneal epithelial healing processes and a greater vulnerability to chronic epithelial defects, along with cornea infections (2, 3). Diabetes-related damage to the corneal nerves reduces trophic support, which leads to increased squamous cell loss and diminished proliferation (4). In addition, diabetes ocular complications include ocular epitheliopathy and adhesion illnesses, as well as alterations in the corneal epithelium’s basal cells and basement membrane (5).

In healthy corneas, “mature” leukocytes were absent due to their limited ability to produce pro-inflammatory cytokines, sensitize lymphoid cells, and trigger effector T cell responses. Instead, “mature” leukocytes advocated the modulation of immunity quiescence and the induction of tolerance to the immune system via the ocular surface (6, 7). However, chronic inflammation and metabolic abnormalities of type 2 diabetes sufferers with hyperglycemia undermined the cornea’s immune privileges. While the changes of immune cells, both in type and quantity, could lead to pathological alterations in diabetic corneas. Immune cells, especially mononuclear cells, exhibited changed traits due to chronic hyperglycemia, such as deficiencies in complement receptors and Fc receptors. These alterations hindered phagocytosis, reduced MHC-II and adhesions, decreased antimicrobial activity and glycolytic capacity, and diminished reserve (8, 9). Moreover, it is widely accepted that diabetes is associated with systemic alterations of adaptive immunity (1). High glucose inhibited lymphocyte recruitment and decreased the production of cell adhesion molecules (CAMs), which included E-selectin and intracellular adhesion molecule (ICAM)-1, hampering the infiltration of CD45+ lymphocytes and CD8+ T cells (10). In addition, regulator T cells (Tregs) in lymph nodes emptying the ocular surface can inhibit both the sensitization of naive T cells and the function of activated T cells, thereby preserving the immune quiescence of the ocular surface (11). Nonetheless, ongoing inflammation and metabolic issues associated with type 2 diabetes reduce the total number of Tregs (12).

Despite extensive research and focus on the function of immune cells within ocular ailments, the atlas of immune cells of diabetic corneas remains elusive. To explore diabetic corneal immune cells, we executed mass CyTOF and devised an immune cell–related antibody panel, which involved 41 immune-related markers. In the corneal tissues of diabetic mice, CD103+CD8+T_RM_ cells and Tregs were significantly decreased, whereas gamma delta T (γδT) and CD11b+Ly6G+ MDSC subset were significantly increased. This investigation offered a comprehensive understanding of the alterations of corneal immune cells within diabetic mice, suggesting the tremendous alterations of corneal immunological microenvironment in a persistent high-glucose status. Through in-depth analysis, it was possible to identify significant immune cell subsets that were relevant to the pathogenesis of diabetic corneal diseases, providing a novel target for treating the disease in the future.

## 2 Materials and methods

### 2.1 Animal model

The db/db and db/m mice with 8 weeks old were bought from Peking University Health Science Center’s Department of Laboratory Animal Science. All experimental procedures were adhered to, according to the National Research Council’s Guide for the Care and Use of Laboratory Animals, and were approved by the Chinese PLA General Hospital’s (Beijing, China) Scientific Investigation Board. In this study, only male mice were used, as female sex steroids safeguard mice from developing diabetes (13, 14). Through the duration of the investigation, mice were feeded in groups of 3–4 per cage, kept on a 12-h light/12-h darkness cycle, as well as had unrestricted access to food and water. At the age of 21 weeks, the db/db mice (DB group) alongside db/m mice (DM group) had been euthanized.

### 2.2 Corneal tissues collection

As a well-known type 2 diabetes models, this DB mouse line has a series of characteristics such as significant increases in body weight beginning at 4 weeks, hyperglycemia at 8 weeks, insulinemia (> 3-fold) at 8 weeks (15). Moreover, the DB corneas showed significant histopathological alterations, with impaired proliferation, decreased innervation at 20-24 weeks (16), lower density of the corneal subbasal nerve plexus and corneal epithelial branches at 13 weeks (17). We therefore selected 21 weeks aged mice as the test animals. Corneas from five mice and nine eyeballs were collected. The conjunctival sac was flushed continuously with 5 ml of 0.5% gentamicin (Sigma, E003632) diluted in normal saline per eye. The eyeballs were removed under sterile conditions, rinsed three times with DPBS solution (Gibco™, 14190-144), and then immersed in DPBS-double antibody solution (penicillin 100 units/ml, streptomycin 100 μg/ml) (Gibco™, 10378-016) for 10 min on ice. The intact cornea was removed along the limbus with autoclaved ophthalmic scissors on an ultraclean table under a stereomicroscope with a cold light source and then rinsed five times with DPBS-double antibody solution (Gibco™, 10378-016) on ice. The corneal tissue was cut into 0.5–1 mm^3^ pieces on ice and temporarily stored on ice.

### 2.3 Preparation of a corneal single-cell suspension

Corneal tissues were placed in DMEM solution (Gibco™, 11320-033) containing 10% FBS (Thermo Fishier, 10091), DispaseI (1mg/ml) (Thermo Fishier, 17018-029), DispaseII (0.2mg/ml) (Thermo Fishier, 17101-015), DispaseIV (0.5mg/ml) (Thermo Fishier, 17104-019), and DispaseV (0.2mg/ml) (STEMCELL, 100-0681), with a solution volume 40 times greater than the tissue volume. Corneal tissues were digested in a sterile petri dish at 37°C for 1.5 h, with manual resuspension of the tissues at 20 min intervals. Subsequently, a trypsin–EDTA mixture (Gibco™, 25200-072) was added for 10 min at 37°C (final trypsin concentration of 0.1%). The digestion was terminated by adding an equal volume of DMEM+10%FBS (DMEM: Gibco™, 11320-033/FBS: Thermo Fishier, 10091). Before and after the termination of digestion, the tissues were gently blown 10 times with a disposable straw. The corneal tissue suspension was filtrated through a 200-mesh pore size nylon mesh (40µm cell sieve) (Falcon®,352340) centrifugated at 300×g for 5 min at 4°C and resuspended with DPBS solution (DPBS+0.04%BSA) (DPBS: Gibco™, 14190-144/BSA: Roche Diagnostics Deutschland GmbH, 10711454001). 0.1ml of cell suspension was stained with one drop of 0.4% trypan blue (Gibco™, 15250061) and stained for 4 min at room temperature. The cells were counted using a cell counter (Thermo Fishier, Countess) while ensuring that the cell diameter was <40 μm and cell viability >80%. The total number of cells was more than 0.05 × 10^6^, and the background of the cell suspension was clean, without a large number of cell agglomerations, debris and impurities, and free of Ca2^+^ and Mg2^+^.

### 2.4 Cytometry by time of flight (CyTOF/mass cytometry) staining and acquisition

According to the manufacturer’s recommendations, pure antibodies were obtained and internally conjugated using MaxPar x8 Polymer Kits (Fluidigm). 1 x 10^6^ cells were stained for 5 min at room temperature with 1 M cisplatin, rinsed with protein-free PBS, and then stained for 30 min at 4°C within a stain solution (PBS comprising 0.5% bovine serum albumin and 0.02% sodium azide). Cells were coated overnight with DNAIntercalator-Ir following fixation. Utilizing the power source Foxp3/Transcription Factor Staining Buffer Set (eBioscience), intracellular staining was performed for 30 min at 4°C, rinsed, and stored at 4°C until acquisition. After twice washing with deionized water (Helios), Ce140, Eu151, Eu153, Ho165, and Lu175-containing EQ normalization beads (Fluidigm) were added, and CyTOF was then acquired. In total, 41 antibodies were selected for mass cytometry (**Supplementary Table 1**).

### 2.5 CyTOF/mass cytometry data analysis

The CyTOF analysis was conducted by PLTTech Inc. (Hangzhou, China). Volumetric facs data were initially debarcoded via a two-dimensional filtration strategy along with mass-tagged barcodes. FlowJo software was then used to manually gate live, singlet, and functioning immune cell populations (BD). The bead normalization approach was applied to normalize the data produced from multiple batches. To identify distinct cell populations across all data, we employed the unsupervised clustering technique X-shift and dimensional reduction with t-distributed stochastic neighbor embedding.

### 2.6 Statistical analysis

Statistical analysis was conducted using GraphPad Prism software. The unpaired Student’s t-tests were employed for comparison between two groups. A *p*-value<was considered statistically significant. (*p<0.05, **p<0.01***p<0.001, and ****p<0.0001).

## 3 Results

### 3.1 Immune signature traits in the corneas of diabetic (db/db) (DB group) mice

**Figure 1:**
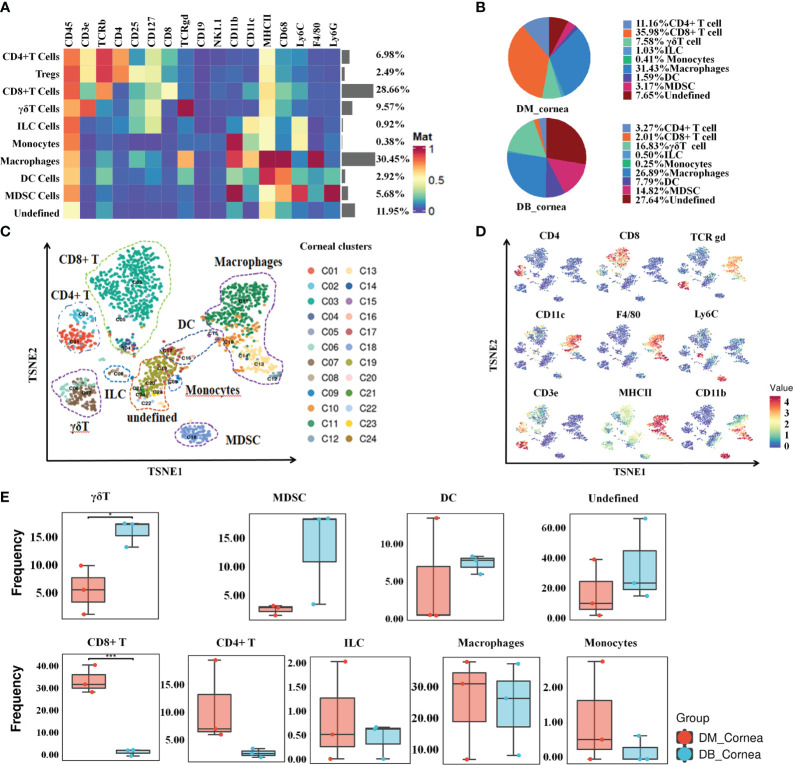
Signature characteristics of corneal immunity in non-diabetic (db/m) and diabetic (db/db) rodents. **(A)** Heatmap analysis of the proportions of corneal main immune cell groups in db/m and db/db mice. **(B)** Pie chart depicting the proportional differences between db/m (DM group) and db/db (DB group) corneal major immune cell subsets. **(C)** TSNE diagram displaying the spatial arrangement of 24 immune cell clusters in corneal tissues of db/m and db/db mice. **(D)** Using the t-SNE algorithm, the main immune cell markers over corneal immune cell subgroups have been analyzed. **(E)** Frequency differences and similarities of corneal immune cell subsets among db/m and db/db mice. *P < 0.05, **P < 0.01, ***P < 0.001.

To characterize the dynamic profile that defines the diabetic corneal immune microenvironment, we performed CyTOF analyses on three pairs of diabetic and non-diabetic corneal samples (each sample containing nine corneal tissues), respectively. As shown in **Figures 1A, B** and **Supplementary Table 2**, 8 main immune cell subsets were identified by distinct signaling antibodies, including CD8+T cells, CD4+ T cells (containing Tregs), γδ T cells, ILC, MDSC, DC, Macrophages and Monocytes (**Figure 1A**). Among them, T cells and macrophages, comprising 47.7% and 30.45% of corneal immune cells, respectively (**Figures 1A, B**), were the predominant immune cells. In non-diabetic corneas, CD8-positive T cells have been the most abundant immune cell subset, accounting for 35.98% (**Figure 1B**). Whereas in diabetic corneas, T cells as well as myeloid-derived suppressor cells (MDSC), constituted 16.83% and 14.88%, respectively (**Figure 1B**). The small subsets of immune cells included dendritic cells (DC), innate lymphoid cells, and monocytes (**Figure 1B**).

As shown in **Figures 1C, D**, the proportions of various immune cell subsets in non-diabetic (db/m) and diabetic (db/db) corneas were visualized through t-SNE analysis. Following T-distributed stochastic neighbor embedding analysis, the main cells were divided into 24 clusters (**Figure 1C**). When compared with the DM group, the proportion of CD8+ T cells was substantially reduced within the DB group, whereas the ratio of γδ T cells increased significantly (**Figure 1E**). In addition, other immune cell subsets did not differ considerably between the DM and DB groups, such as CD4+ T, ILC, MDSC, DC, Macrophages, Monocytes (**Figure 1E**).

### 3.2 Immunological heterogeneity of T cell subsets within the DB group

**Figure 2:**
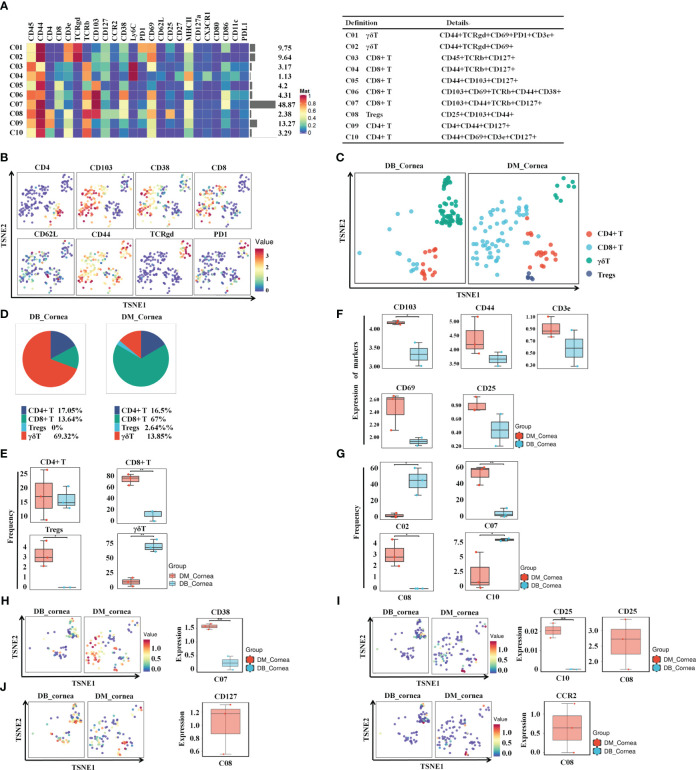
Identifying the immune heterogeneity of corneal T cell subsets. **(A)** Heatmap displaying the general proportions of corneal primary T immune cell subsets (left) as well as definitions of T cell subsets (right) in db/m and db/db mice. **(B)** Functional trait distributions occur in the various main subsets of T cells. **(C)** A TSNE plot depicting the distribution of T cell subsets. **(D)** Pie chart depicting the proportional differences between db/m (DM group) as well as db/db (DB group) primary T cell subsets. **(E)** Frequency differences and similarities between the non-diabetic(db/m)(DM group) and db/db(DB group) T cell subsets. **(F)** Development of CD8T cell surface markers (CD103,CD44,CD3e,CD69,CD25) within corneal tissues of diabetic (db/db) and non-diabetic (db/m) mice. CD103 differences were statistically significant. **(G)** Significant differences between non-diabetic (db/m) (DM group) as well as diabetic (db/db) (DB group) T cell subsets. **(H)** CD38, **(I)** CD25, **(J)** CD127, and **(J)** CCR2 in specific T cell subsets of db/m and diabetic (db/db) mice. *P < 0.05, **P < 0.01, ***P < 0.001.

To discover the distinctive characteristics of T cells in the pathogenesis of diabetic corneal complications, we conducted a single-linkage clustering analysis between the DM and DB groups concentrating on CD44+CD127+ T cell subsets (**Figures 2A, B**). As a result, 4 clusters were obtained (**Figure 2A**). Based on the expression of the specific markers, they were grouped into γδT cells, CD8+ T cells, CD4+ T cells, and CD25+CD103+ Tregs (**Figure 2A**). Immune cells from all samples analyzed by T-SNE and colored according to the relative expression of CyTOF markers (CD4, CD103, CD38, PD1, TCRgd, CD8, CD62L, CD44) (**Figure 2B**). When compared to the DM group, the proportion of Tregs and CD8+ T subgroups in DB group was significantly decreased, while the percentage of γδ T cells expanded significantly (**Figures 2C–E**). Among them, γδ T cells and CD4+ T, comprising 69.32% and 17.05% of corneal immune cells from DB mice, respectively (**Figure 1D**), were the predominant immune cells. On the contrary, CD8+ T (67%) and CD4+ T(16.5%) were the primary immune cells of corneal immune cells from DM mice (**Figure 1D**). Moreover, CD4+ T cells showed no significant change between the DB and DM groups (**Figure 2E**). The expression of CD103 in the surface marker of corneal CD8+ T cells (CD103, CD44, CD3e, CD69, and CD25) was statistically significant in corneal tissues of both DM group and DB group mice (**Figure 2F**).

In particular, the percentage of C07 (CD8+ T cell) and C08 (Tregs) in the DB group was significantly reduced. **Figure 2G** demonstrates that the percentage of C02 (γδ T) along with C10 (CD4+ T cell) was substantially greater throughout the DB cohort compared with the DM group. The aforementioned findings indicate that the changes in the type and number of T cell subsets in the DB group may contribute to the pathological alterations of corneal structures in diabetic corneas. **Figures 2H, I** demonstrate that the levels of CD38 in the subsection of the C07 subset decreased substantially in the DB group, whereas the level of CD25 decreased significantly in the subgroups of C08 and C10 (**Figures 2H, I**). CD127 and CCR2 levels within the C08 subgroup within the DB group were substantially lower than those of the DM group (**Figure 2J**).

### 3.3 Immunological heterogeneity of myeloid cells in the DB group

**Figure 3:**
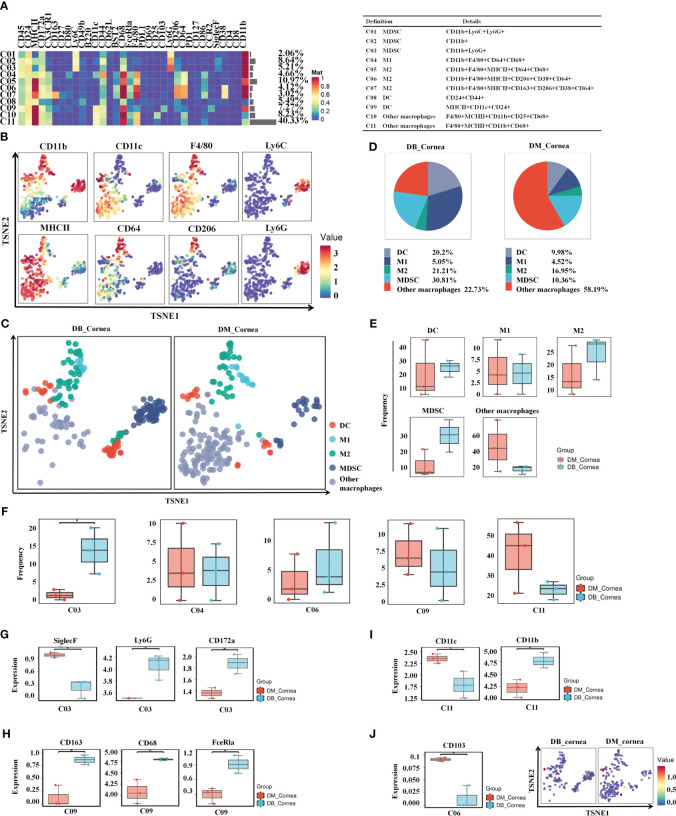
Identification of heterogeneity in corneal immune myeloid cell subsets. **(A)** Heatmap displaying the general proportions of corneal main myeloid cell subsets (left) as well as the definitions of myeloid cell subsets (right) in db/m and db/db mice. **(B)** Various myeloid cell subsets express distinct distributions of main functional characteristics. **(C)** A TSNE plot displaying the distribution of myeloid cell subsets. **(D)** Pie chart depicting the proportional differences between non-diabetic (db/m) (DM group) as well as diabetic (db/db) (DB group) primary myeloid cell subsets. **(E)** Frequency differences between non-diabetic (db/m) (DM group) as well as diabetic (db/db) (DB group) myeloid cell subsets. **(F)** Significantly altered or unaffected myeloid cell groups among db/m mice and db/db mice. **(G)** SiglecF,Ly6G,CD172a, **(H)** CD163,CD68,FceRla, **(I)** CD11c,CD11b, and **(J)** CD103 in particular T cell subsets of db/m as well as db/db rodents. *P < 0.05, **P < 0.01, ***P < 0.001.

After re-clustering analysis, the myeloid cell population was grouped into 11 clusters (**Figure 3A**). Using the characteristic markers CD11b, F4/80, CD11c, MHCII, CD64, and CD206, we compared the differences in myeloid cell expression among the DB and DM groups (**Figure 3B**). The myeloid populations were merged into 5 main immune cell subsets, including MDSC, M1-Macrophages, M2-Macrophages, other Macrophages and DC (**Figure 3C**). Among them, MDSC and other Macrophages, comprising 30.81% and 22.73% of corneal immune cells from DB mice, respectively (**Figure 3D**), were the predominant immune cells. On the contrary, other Macrophages (58.19%) and M2-Macrophages (16.5%) were the primary immune cells of corneal immune cells from DM mice (**Figure 3D**).

The vast majority of myeloid cells did not differ significantly between the DB and DM groups, such as MDSC, M1-Macrophages, M2-Macrophages, other Macrophages and DC (**Figure 3E**). In particular, the C03 (MDSC) ratios were considerably higher compared to the DB group (**Figure 3F**). SiglecF was significantly reduced between the C03 subgroups within the DB group, whereas Ly6G and CD172a expressions were substantially increased (**Figure 3G**). Meanwhile, the expressions of CD11c in the C11 subgroups and CD103 in the C06 subset were significantly decreased, whereas CD163, CD68, and FceRla in the C09 subset and CD11b in the C11 subset were markedly higher within the DB group versus the DM group (**Figures 3H–J**).

## 4 Discussions

Diabetes causes significant alterations in the composition and function of immune cells in the cornea. These changes result in a variety of diabetic corneal complications, including postponed corneal epithelial wound healing, recurrent erosions, neuropathy, loss of sensitivity and tear film alterations (18). Despite being an avascular tissue, the cornea is hyperglycemic sensitive. Relatively little is known about immune microenvironment alterations in diabetic corneas. To dissect the immunological change of diabetic corneas, 41 immune cell markers were selected for CyTOF/mass cytometry on corneal tissues from diabetic and control mice. In this study, we identified several alterations of immune cell subpopulations, characterized by notable decreases in CD8+T_RM_ cells and Tregs, as well as significant increases of γδT cells and CD11b+Ly6G+ MDSCs.

Although the cornea is considered as immune-privileged tissue, ocular infection results in the formation of CD103+CD8+T_RM_ cells, which patrol the cornea and provide local protective immunity in healthy corneas (19). These cells mediate ex vivo cytotoxicity or clear pathogens through non-cytolytic approaches (19, 20). In our study, we revealed reduced CD103+ CD8+ TRM cells in diabetic corneas, as well as lowered expression of CD103. Accumulative evidence showed that corneal CD103+ T_RM_ formation requires transforming growth factor β (20). CD103 has also been linked to the initial accumulation of effector CD8+ T cells in tissue (21). Until now, there have been few studies on TRM cells from the corneas of diabetic mice. Two subsets of TRMs and four subsets of recirculating T cells were proved to protect the human ocular surface, one of which was CD103+CD8+T_RM_ cells. This results was more akin to gastric mucosa than skin, lung, intestine, or cervix (22).TRMs in the cornea were heterogeneous for expression of CD103, suggesting that cells reach the corneal epithelium could stimulate CD103 (19, 23). Further studies are required to investigate the association of reduced CD8+ TRM with diabetic corneal disorders, as well as their underlying mechanisms.

One of the most surprising discoveries in the current investigation is the significant decrease in Tregs and the remarkable increase of γδT cell subsets in the DB group compared to the DM group. As immunosuppressive cells, Tregs play essential roles in resolving excessive immune responses and maintaining homeostatic tolerance (24). Adoptive transfer of Tregs has been proven to be effective in protecting against various autoimmune and autoinflammatory diseases, such as experimental autoimmune encephalomyelitis (25), experimental autoimmune uveitis (26) and dry eye disease (27). In type 1 diabetes (T1D) and T2D settings, the dysfunction of Tregs is an important mechanism for the pathogenesis of diabetes (28). Adoptive transfer of Tregs could effectively alleviate the symptoms (29) mechanistically by inhibiting effector T cell proliferation and providing anti-inflammatory effects (30). For the first time, we showed a significant reduction in Treg frequency in diabetic corneas, but its associations with diabetic corneal complications remain unclear. Based on the nature of Tregs in the maintenance of immune homeostasis, the lowered Treg frequency probably contributed to the chronic inflammation of diabetic corneas. Moreover, tissue-resident Tregs have been reported to modulate tissue homeostasis and regeneration, including intestinal stem cells (31), muscle stem cells (32) and corneal limbal epithelial stem cell (33). In this regard, the reduced Tregs probably promoted pathological alterations of diabetic corneas by impairing the functioning of limbal epithelial stem cells.

As a unique T lymphocyte subpopulation, γδ T cells form an important component of adaptive immunity in protecting against infection and malignant transformation (34, 35), and maintaining tissue homeostasis (36–38). A lack of γδT cells has been linked to exacerbated autoimmune responses and increased sensitivity of epithelial or mucosal tissues to injury (39–41). Growing evidence indicates that the γδT cells protect the corneas against infection (42, 43) under physiological conditions but worsen disease development under some pathological scenarios, including allergic conjunctivitis (44) and dry eye disease (45). Although we showed an increased frequency of γδ T cells in diabetic corneas, little is known about their roles in diabetic corneal pathological alterations. The exact roles of γδ T cells in the development of diabetes remain complex, depending on different γδ T cell subsets (46, 47). Therefore, to comprehend the functions of γδT cells in diabetic corneal diseases, the functional properties of γδT cells under diabetic conditions should be fully determined in future investigations.

Among myeloid cell subsets, the increased ratio of MDSC subset (C03) in the diabetic cornea was observed, with elevated expression of SiglecF, Ly6G, and CD172a. MDSCs with potent immunosuppressive functions consist of two major groups: granulocytic and monocytic (G/M-MDSC) populations (48). MDSCs are reported to be implicated in immunomodulation in numerous pathological settings, such as tumerigenesis (48, 49), autoimmune diseases (50, 51) and chronic infection (52, 53). Although several findings have documented important roles of MDSCs in ocular diseases, including herpetic stromal keratitis (54) and corneal transplantation rejection (55, 56), the exact role of MDSC in diabetic corneal pathology has not been explored. In diabetes mellitus, alterations in MDSC cell numbers and their immunosuppressive activity vary in different organs and tissues (57, 58). Based on the immunosuppressive properties, the increased MDSC subset probably contributes to the chronic inflammatory state of the diabetic cornea. However, future investigations are required to determine in detail their function in diabetic corneas.

In this study, we provided an atlas of immune microenvironments in diabetic corneas. Nonetheless, there are also several limitations to address in future. First, the immune cell subpopulations identified in diabetic corneas should be validated in a larger sample size through experimental approaches, including immunofluorescence staining. Second, in addition to the change in immune cell frequency, functional alterations must be investigated in diabetic conditions. Finally, the role of certain immune cell subpopulations in the development of diabetic corneal pathological alterations should also be explored.

## 5 Conclusions

In conclusion, CyTOF analysis revealed significant alterations of the immunological microenvironment during the development of diabetic corneal disorders, manifested with a notable decrease in CD8+ TRM cells and Tregs and a dramatic increase of γδ T Cells and CD11b+Ly6G+ MDSC subsets. This study mapped the immune microenvironment landscape of type 2 diabetic corneas, providing new insightful guidance for investigating the pathogenesis of diabetic ocular surface complications.

## Data availability statement

The datasets presented in this study can be found in online repositories. The names of the repository/repositories and accession number(s) can be found below: Huang, Yifei; Qin, Limin; Li, Qian (2023), “diabetic cornal immune cells”, Mendeley Data, V1, doi: 10.17632/j5hb3n5wnv.1. https://data.mendeley.com/datasets/j5hb3n5wnv/1.

## Ethics statement

The animal study was approved by Chinese PLA General Hospital’s (Beijing, China) Scientific Investigation Board. The study was conducted in accordance with the local legislation and institutional requirements.

## Author contributions

LQ and QL conceptualized and performed the experiments, analyzed and interpreted the data, and helped write the manuscript. LQ and QL performed the experiments helped analyze and interpret the data. YH and LW conceptualized the experiments, secured funding, interpreted the data, and supervised the research activity. All authors contributed to the article and approved the submitted version.
