# Supplementary material for: Mass cytometry reveals the corneal immune cell changes at single cell level in diabetic mice
Source: Front Endocrinol (Lausanne). 2023 Sep 5;14:1253188. doi: 10.3389/fendo.2023.1253188 (PMC10507693; doi:10.3389/fendo.2023.1253188)
Supplement: Supplementary file 1 [file Table_1.docx]

Supplementary Table 1 Panel design of CyTOF for this study

| [**sequence number**](javascript:;) | [**antibody**](javascript:;) | [**sequence number**](javascript:;) | [**antibody**](javascript:;) |
| --- | --- | --- | --- |
| 1 | CD45 | 22 | TCRb |
| 2 | CD3e | 23 | PDL1 |
| 3 | CD24 | 24 | CD69 |
| 4 | MHCII | 25 | CD25 |
| 5 | CD172a | 26 | CD103 |
| 6 | CX3CR1 | 27 | NK1.1 |
| 7 | CD163 | 28 | Ly6G |
| 8 | CD27 | 29 | CD206 |
| 9 | CD80 | 30 | CD64 |
| 10 | Ly6C | 31 | P2RY12 |
| 11 | CD19 | 32 | IgD |
| 12 | CD49b | 33 | PD1 |
| 13 | B220 | 34 | CD127 |
| 14 | CD11c | 35 | CD86 |
| 15 | CD44 | 36 | CCR2 |
| 16 | CD62L | 37 | SiglecF |
| 17 | BST2 | 38 | CD38 |
| 18 | CD68 | 39 | CD4 |
| 19 | FceRIa | 40 | CD8 |
| 20 | TCRgd | 41 | CD11b |
| 21 | F4_80 |  |  |
