# Supplementary material for: Mass cytometry reveals the corneal immune cell changes at single cell level in diabetic mice
Source: Front Endocrinol (Lausanne). 2023 Sep 5;14:1253188. doi: 10.3389/fendo.2023.1253188 (PMC10507693; doi:10.3389/fendo.2023.1253188)
Supplement: Supplementary file 2 [file Table_2.docx]

Supplementary Table 2 The standard for defining corneal immune cells

| cluster/sort merge1 merge2 classification1 classification2 classification3 marker expression |
| --- |
| C01 CD4+T T cells CD4+T Tem CD3+CD4+CD44+CD62L-CD127+ |
| C02 CD4+T T cells CD4+T/Tregs CD103+Tregs CD3+CD4+CD44+CD103+CD25+CD127low |
| C03 CD8+T T cells CD8+T CD103+ CD3+CD8+CD44+CD103+CD38+CD127+ |
| C04 CD8+T T cells CD8+T CD3+CD8+CD44+CD127+CD62L- |
| C05 CD8+T T cells CD8+T CD103+ CD3lowTCRblowCD8lowCD69+CD103+CD127+ |
| C06 γδT T cells γδT CD103+ CD3+TCRgd+CD44+CD69+CD103+PD1lowCD38- |
| C07 γδT T cells γδT CD3+TCRgd+CD44+CD69+CD103-PD1+CD38+ |
| C08 ILC ILC CD45+MHCⅡ+CD103-CD68-CD69+CD38+CD127+ |
| C09 Monocytes Monocytes Ly6C+CD11b+ |
| C10 Macrophages M1-like Macrophages M1-like CD11b+F4/80+CD68+MHCⅡ+CD86+ |
| C11 Macrophages M1-like Macrophages M1-like CD11b+F4/80+CD68+MHCⅡ+CD86+ |
| C12 Macrophages M2-like Macrophages M2-like CD11b+F4/80+CD68+MHCⅡ+CD163+CD206+CD38+ |
| C13 Macrophages Macrophages other macrophages CD11b+F4/80+CD68+MHCⅡ+CD206+ |
| C14 Macrophages Macrophages other macrophages CD11b+F4/80+CD68+MHCⅡ+CD64+ |
| C15 DC DC CD103+DC MHCⅡ+CD11c+CD103+ |
| C16 DC DC pDC MHCⅡ+CD44+CD24+BST2+B220+ |
| C17 DC DC MHCⅡmidCD24midCD44mid |
| C18 MDSC MDSC CD11b+Ly6G+Ly6C+ |
| C19 Undefined Undefined CD45mid |
| C20 Undefined Undefined CD45mid |
| C21 Undefined Undefined CD45mid |
| C22 Undefined Undefined CD45mid |
| C23 Undefined Undefined CD45mid |
| C24 Undefined Undefined CD45mid |
|  |
